# Design, Synthesis, and In Vitro Evaluation of Novel Indolyl DiHydropyrazole Derivatives as Potential Anticancer Agents

**DOI:** 10.3390/molecules26175235

**Published:** 2021-08-29

**Authors:** Katharigatta N. Venugopala, Mohammed Habeebuddin, Bandar E. Aldhubiab, Afzal Haq Asif

**Affiliations:** 1 Department of Pharmaceutical Sciences, College of Clinical Pharmacy, King Faisal University, Al-Ahsa 31982, Saudi Arabia; baldhubiab@kfu.edu.sa; 2 Department of Biotechnology and Food Technology, Durban University of Technology, Durban 4001, South Africa; 3 Department of Biomedical Sciences, College of Medicine, King Faisal University, Al-Ahsa 31982, Saudi Arabia; Hmohammed@kfu.edu.sa; 4 Department of Pharmacy Practice, College of Clinical Pharmacy, King Faisal University, Al-Ahsa 31982, Saudi Arabia

**Keywords:** AhR inhibitors, indoles, pyrazolines, anti-proliferative, molecular docking

## Abstract

Indoles derived from both natural sources or artificial synthetic methods have been known to interact with aryl hydrocarbon receptors (AhR), and exhibit anticancer activity. In light of these attractive properties, a series of hybrid molecules with structural features of indoles, i.e., those bearing a pyrazoline nucleus, were evaluated for their enhanced anticancer activity. The designed molecules were subjected to molecular docking in order to screen for potential AhR interacting compounds, and the identified indolyl dihydropyrazole derivatives were synthesized. The synthesized compounds were characterized, and their cytotoxicity was evaluated against four human cancer cell lines using the MTT assay. Based on the Glide g-score, H-bonding interactions and bonding energy of 20 candidate molecules were selected for further analysis from the 64 initially designed molecules. These candidate molecules have shown promising anti-proliferative activity against the cell lines tested. Among these candidate molecules, the compounds with hydroxy phenyl substitution on the pyrazoline ring have shown potent activity across all the tested cell lines. The designed scaffold was proven effective for screening potential candidate molecules with anticancer properties, and may be further optimized structurally for yielding the ideal anti-tumorigenic compound for the treatment of various cancers.

## 1. Introduction

The aryl hydrocarbon receptor (AhR) is a cytosolic regulatory protein of the Per–ARNT–Sim (PAS) protein family, and can be stimulated by several endogenous and exogenous ligands. The AhR, in the absence of a ligand, is present in the cytoplasm as a tetrameric chaperone complex, which includes the dimer of Hsp90, p23, immunophilin-like X-associated protein 2 (XAP2), and AhR-interacting protein (AIP) [1,2,3]. Ligands binding to the complex induces structural changes, which in turn results in the dissociation of the chaperone complex, exposing the nuclear localization sequence which enables its translocation towards the nucleus along with the ligand [4,5]. The AhR nuclear transporter (ARNT) association promotes the formation of the high-affinity DNA-binding complex, a complex of heterodimeric transcription factor [6]. The formation of the AhR/ARNT heterodimer enables it to bind to the DNA replication-related element (DRE) of the DNA sequence, which in turn leads to the remodeling of the chromatin network triggering transcription of downstream genes [7,8,9]. The AhR has been reported to regulate various biological pathways, and play a major role in the maintenance of normal cellular functions, which includes maintaining intestinal homeostasis, epithelial barrier function, host immune function & regulation, epidermal formation, organogenesis, cell migration, cell cycle, and proliferation [10,11,12,13,14].

Aberrant AhR expression often leads to functional anomalies, and is the underlying reason for cancer, as evident from the various in vitro and in vivo models of cancer, with noticeable divergences in pro- and anti-tumorigenic profiles. Moreover, different classes of ligands modulate the AhR functionality, and ligands of the same class in fact have been reported to produce different tumorigenic outcomes by influencing the AhR functionality. However, AhR is considered as a potentially critical drug target, which has the potential to be modulated by several classes of ligands [15].

The activation of AhR regulates target genes controlling inflammation, which includes IL-6, IL-22, PTGS2, VEGFA, and CYP1A1, and acts as an important regulator of immunity and inflammation [16,17]. Hence, modifications in the AhR expression or its activation may lead to significant adverse health outcomes. The activation of AhR by indoxyl sulfate (IS) has been described to modulate IL-6 and various CYP enzymes (CYP1A1, CYP1A2, CYP1B1) which play an important role in regulating the inflammation in proximal tubular cells [18,19,20]. The agonists of AhR can modulate the differentiation of certain T cells and recent studies have reported indole-3-pyruvic acid (IPA) to promote the differentiation of Tr1 cells, resulting in an increase in IL-10 production which in turn modulates the inflammatory and immune homeostasis in the intestine [21,22].

6-Formylindolo (3,2-b) carbazole (FICZ), a photoproduct of tryptophan, can induce AhR activation which mediates the inhibition of inflammation by antagonizing the effect of IL-6 in lipopolysaccharide-treated dendritic cells and dextran sulphate sodium-induced colitis [23,24,25]. FICZ was reported to establish FICZ/AHR/CYP1A1-dependent negative feedback mechanism to regulate active cellular processes [26]. Another metabolite of tryptophan, 3,3′-diindolylmethane (DIM), which is a selective AhR modulator, has been reported to inhibit AhR/ARNT-dependent apoptosis and autophagy, and plays an active role in protecting the hippocampal cells against hypoxia [27]. The activation of AhR by indole-3-carbinol (I3C) initiates the chain reactions involving Rbx1 E3 ubiquitin ligase which promotes the ubiquitination of ERα and eventually leads to the loss of ERα-mediated proliferation [28]. 

Indole derivatives were reported to interact with various intracellular targets, including AhR and cyclin-dependent kinases (CDKs), and were found to exhibit anticancer activity. In particular, indolocarbazoles obtained from natural and synthetic origin have been shown to have anti-proliferative activities in different cell lines [29,30].

Based on previous studies involving indoles and pyrazolines, it may be hypothesized that a hybrid molecule having structural characteristics of both indoles and pyrazolines may present a substantial improvement in the anti-proliferative activity of the indole derivatives. In this purview, our group designed potential compounds with indole moiety attached at the third position of the pyrazoline ring with subsequent substitution of the pyrazoline nitrogen atom, resulting in the formation of a 5-(indol-3-yl)-pyrazoline-1-carboxamide derivative. The indole-pyrazoline hybrid compounds were designed and screened by in silico approaches, and the selected molecules were synthesized and evaluated for potential anti-proliferative activity.

## 2. Materials and Methods

### 2.1. Molecular Modeling Studies

#### 2.1.1. Ligand Preparation

The designed molecule structures were drawn using ChemDraw 18.2 (ChemOffice 2018, Perkin-Elmer Informatics, Shelton, CT, USA), and the files were saved in a .sdf format. The Chem3D 18.2 software was used to minimize the energy of the chemical structure of each compound, such that the energy difference between the bonds was 0.001 kJ/mol, and a single minimum energy 3D conformation of the compound was subsequently generated. These 3D structure files were opened using Maestro 11.2 (Schrodinger, LLC, New York, USA), and ligand preparation was performed using the LigPrep tool [31], with a filtration criteria of molecular weight of ≤150 and ≥500, and force field OPLS3. The default settings were maintained with the following options: ionization; generate possible states at target pH 7.0 ± 2.0; Epik program for ionization states; desalt; generate tautomers; stereoisomers: retain specified chiralities; stereoisomers; generate at most 32 per ligand; output format: maestro. The LigPrep results were verified, and one conformation per ligand was selected based on their Epik state penalty (in kcal/mol) [32].

#### 2.1.2. Protein Preparation

The crystal structure of the PAS-A domain of AhR (PDB ID: 4M4X) [33] was imported into a Maestro protein preparation tool from RCSB PDB, and the protein structure was modified under more stringent criteria in terms of integrity, and missing residues were included using the prime program. Later hydrogen atoms were added to the protein and optimization and minimization of the energy of the protein was evaluated. The H-bond assignment section of the software was used for optimizing the hydrogen bonding network, a process in which the samples’ orientation can be altered and Asn, Gln, and/or His side chains can be flipped at a specified pH value. The protein protonation states were maintained under the pH range of 7.0 ± 2.0, and the geometry of the chemical structure was optimized with a RMSD maxima of 0.3 Å using the OPLS3 force field [34,35].

#### 2.1.3. Active Site Prediction

The protein was studied and possible binding sites were characterized using the SiteMap tool [36]. SiteMap analysis highlights the regions surrounding the binding sites, which are potentially convenient for docking the hydrophobic functional units or ligand H-bond acceptors, donors, or metal-chelating functional groups. Out of the five binding sites predicted by the Sitemap, one binding site was chosen for further docking investigations [37,38].

#### 2.1.4. Receptor Grid Generation

Around the chosen binding site of the protein 4M4X, a grid was generated by using the Glide program by selecting one residue in the binding cavity with a grid box of 16×16×16 Å, and a length of 8 Å corresponding to the x, y, and z coordinates of 11.55, −8.28, and 8.78, respectively. With these coordinates and default parameters, the grid file was generated and saved for further docking studies [39].

#### 2.1.5. Molecular Docking Study

In the Glide program, flexible docking was employed to identify the possible binding interactions, and affinity between the designed molecules and the predicted binding site of protein 4M4X. The generated receptor grid files were opened using the Glide program, and the prepared ligands from the workspace were included to study the binding interaction with the protein 4M4X using the standard precision (SP) docking program with all default parameters unaltered [34,35,40,41].

### 2.2. Synthesis of Pyrazoline Derivatives of Indole

All the chemicals were purchased from Sigma-Aldrich, and all the solvents for the use were of laboratory grade. The materials for in vitro evaluation were obtained from Sigma-Aldrich Ltd. The reactions were monitored by thin layer chromatography (TLC) on precoated silica gel 60 F254 (Merck, Darmstadt, Germany), and spots were visualized with UV light. Merck silica gel (80–120 mesh; Merck, Darmstadt, Germany) was used for column chromatography. All the synthesized compounds were purified by recrystallization and column chromatography. Melting points were determined via an open capillary method and were uncorrected. IR spectra were recorded on a Perkin-Elmer FT-IR 240-C Spectrophotometer using KBr optics (Perkin-Elmer, Waltham, MA, USA). Next, 1H-NMR spectra were recorded on Gemini Varian (Varian, Palo Alto, CA, USA) 200 MHz, Bruker (Bruker Bioscience, Billerica, MA, USA) AV 300 MHz, and Unity (Varian) 400 MHz spectrometer in DMSO-d6 or CDCl3 using TMS as an internal standard. Electron impact (EI) and chemical ionization mass spectra were recorded on a VG 7070 H instrument (Micromass, St Leonards-0n Sea, UK) at 70 eV.

#### 2.2.1. One-Pot Synthesis of Pyrazolines (3a–k)

**Figure 1:**
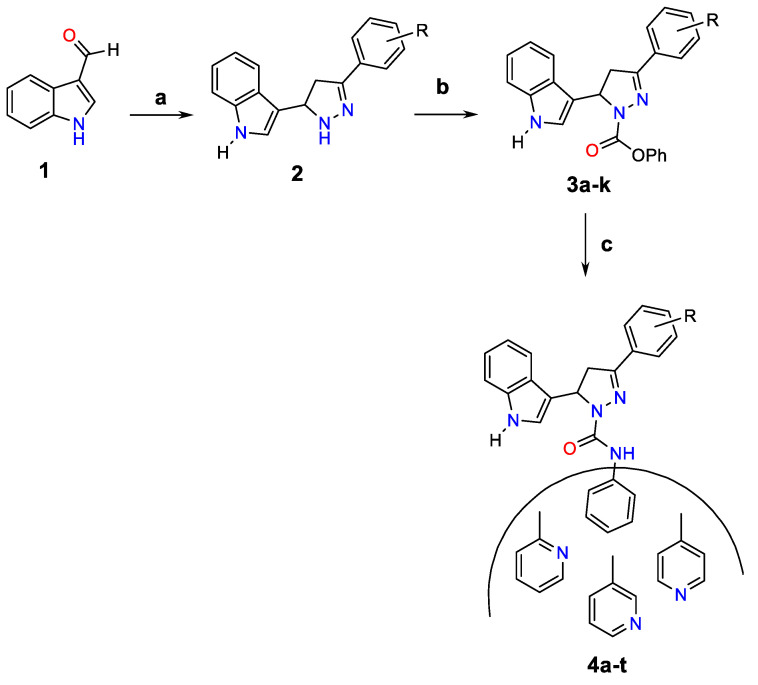
Scheme for the synthesis of indolyl pyrazolines. **a.** NaOH, NH_2_NH_2_, 6–8 h, reflux, **b.** THF, (Et)_3_N, phenyl chloroformate, 2h, below 5 °C, **c.** Ar-NH_2_, RT, stirring, 3–4 h.

To a mixture of indole-3-aldehyde (1 mM) and substituted acetophenone (1 mM), sodium hydroxide (4%, 3 mM) and hydrazine (1.5 mM) were added in 25 mL of ethyl alcohol (96% *v*/*v*), and the resultant mixture of reaction was subjected to heating under reflux with constant stirring for about 6–8 h (Figure 1). The reaction completion was monitored by TLC and the color change was assessed. After confirmation of completion of the reaction, the reaction mixture was allowed to cool to room temperature and kept in the freezer section of the refrigerator overnight (<0 °C). The resultant solid produced was filtered under vacuum, and washed with cold water several times, oven dried, and recrystallized using methanol as the solvent to obtain the pyrazolines (3a–t) [42,43].

#### 2.2.2. Procedure for the Synthesis of 5-(1H-Indol-3-yl)-3-substituted-N-(pyridin-2-yl)-4,5-dihydro-1H-pyrazole-1-carboxamide (4a–t)

Appropriate amounts of pyrazoline derivatives (1 mM) dissolved in dry tetrahydrofuran were added to a mixture of phenyl chloroformate (1 mM) and triethylamine (1 mM) in dry tetrahydrofuran, and an ice-cool condition was maintained. The resultant reaction mixture was stirred continuously for 2 h at temperatures below 5 °C (Figure 1). The resultant solid was removed by filtration and aryl/heteroaryl amine was added to the filtrate, followed by stirring for about 3–4 h at room temperature. The obtained solid was separated, dried, and recrystallized in suitable solvent.

### 2.3. Anti-Proliferative Activity

#### 2.3.1. Cell Culture

Human tumor cell lines (provide by National Centre for Cell Science (NCCS)), Pune, India.), i.e., A-549 (non-small cell lung cancer), MCF-7 (mammary gland adenocarcinoma), Hep G-2 (liver cancer), and DU-145 (prostate cancer) cells, were grown in RPMI 1640 medium or DMEM medium enriched with Glutamax-I and glucose. Both the cell culture medias were supplemented with 10% (*v*/*v*) fetal calf serum, antibiotics penicillin (100 IU/mL), and streptomycin (100 µg/mL), and maintained at 37 °C. The cell viability test was performed before each assay using the trypan blue exclusion method and assays were performed with cells showing <95% viability.

#### 2.3.2. Cytotoxicity Assay

The IC_50_ values of the compounds were calculated by a non-linear regression model with the help of normalized dose–response data acquired by the MTT assay [44,45]. The cytotoxic activity of tested compounds was evaluated using the cell-based spectrophotometric assay with an MTT reagent. Briefly, 5 × 10^3^ cells were transferred to each well of a 96-well plate (Sigma-Aldrich, Mumbai, India) and 100 µL of the culture medium was added with the tested compounds at the concentrations of 1–100 µmol/L. The cells were incubated for 72 h, and then 10 µL of MTT (5 mg/mL) (Sigma-Aldrich, Mumbai, India) was added to each well. The plates were incubated for 4 h at 37 °C. During this period, insoluble formazan was produced, which was then dissolved by the addition of 100 µL of 10% sodium dodecyl sulfate into each well and kept aside for an additional 12 h for the dissolving the formazan. The absorbance of each plate was read at 540 nm and 630 nm reference wavelength using the Epoch Microplate Spectrophotometer (Biotek Instruments Inc., VT, Winooski, VT, USA). The blank absorbance was considered as the control, and the readings were expressed as percentage of the control.

## 3. Results and Discussion

### 3.1. Molecular Modeling Studies

AhR is being extensively studied due to its role as an intermediary of various carcinogens from environmental pollutants, and ideal AhR binding ligand 2,3,7,8-tetrachlorodibenzo-p-dioxin (TCDD) was identified for its tumor eliciting properties in several models. However, the function of AhR and AhR ligands have not been widely explored in tumor cells. In this study, we selected the AhR PAS-A domain, with the resolution of a 2.55 Å crystal structure of the protein (PDB ID: 4M4X) for preliminary docking analysis. Based on the structural analysis of the indole compounds having a binding affinity towards AhR, around 64 novel molecules were designed as having a basic structure of 5-(indol-3-yl)-4,5-dihydro-1H-pyrazole-1-carboxamide with different aryl substitutions on the pyrazoline ring, and an aryl/heteroaryl amino group for amide formation (Supplementary Data). The structures were drawn and minimized in 3D, and later subjected to LigPrep for obtaining potential ligand designs for molecular docking. During ligand preparation, structures were excluded based on their molecular weight (>500 Da). The chosen structures were forwarded for docking calculations. All the structures designed were well under 500 Daltons, so all of them were considered for the molecular docking study.

The crystal structure of the PAS-A domain of AhR with PDB ID: 4M4X was retrieved from the RCSB-PDB. The structure of the protein comprises of PER-ARNT-SIM (PAS) domains of the AhR–ARNT complex. The protein data were entered into protein preparation wizard for refinement. The H-bond assignment section was used for optimizing the hydrogen bonding network, a process by which the sample’s water affinity may be changed by flipping Asn, Gln, and/or His side chains at a specified pH value. The pH adjustment changed the protonation states of residues and ligands accordingly, and is useful for determining the experimental conditions. The restrained minimization section was used to fix the clashes that may occur with adding hydrogens or filling missing sidechains. By default, an RMSD of 0.3 Å was used, which minimized both the hydrogens and heavy atoms using harmonic penalty constraints. As there was no active co-crystallized ligand, the SiteMap tool of the Maestro was employed for detecting the potential binding sites based on the protein parameters. It yielded five binding sites, in which the top scored binding site was that of the AhR–ARNT protein–protein interaction domain with a score and volume of 1.253 and 193.109, respectively, which were subsequently omitted. Later, the binding site was chosen based on the score and volume, as well as the hydrophobic and hydrophilic contributions and information of binding residues based on pre-existing literature.

**Figure 2:**
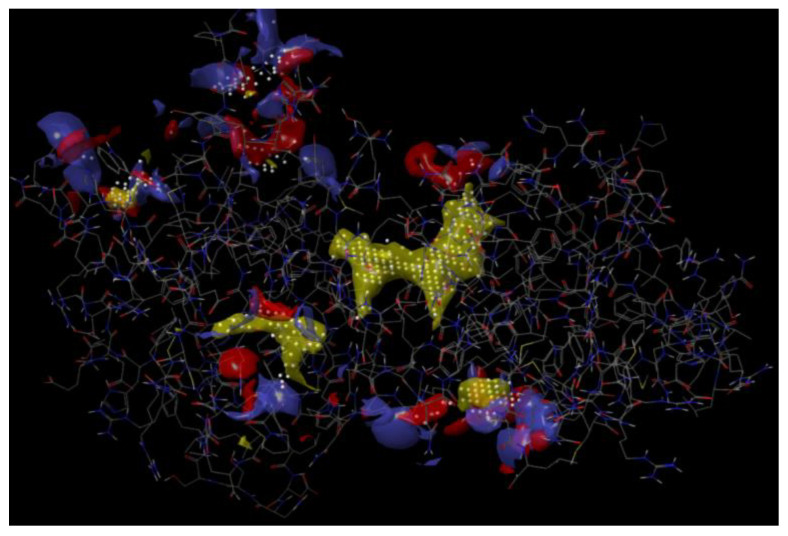
Detection of binding sites using Sitemap tool.

**Figure 3:**
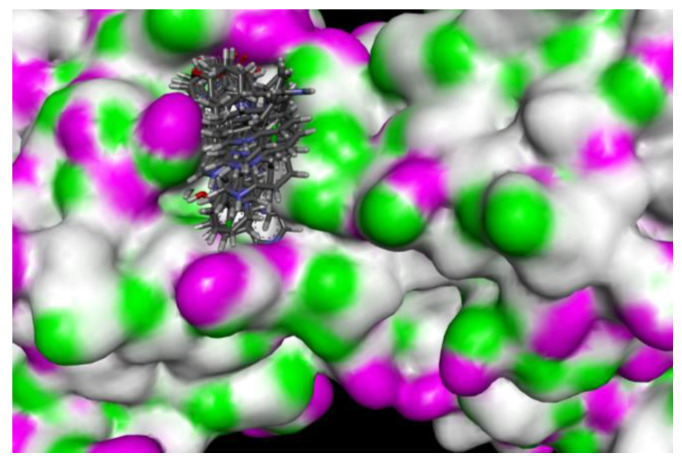
Docked molecules poses in the binding site of the AhR–ARNT complex.

**Figure 4:**
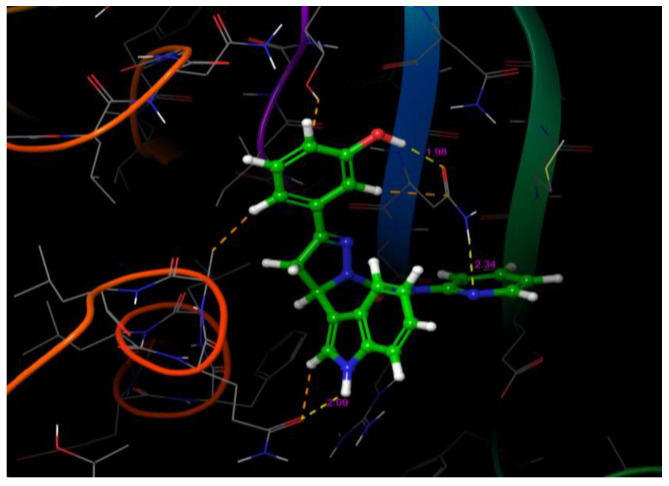
H-bonding interactions of compound after 4h with the binding site residues.

**Figure 5:**
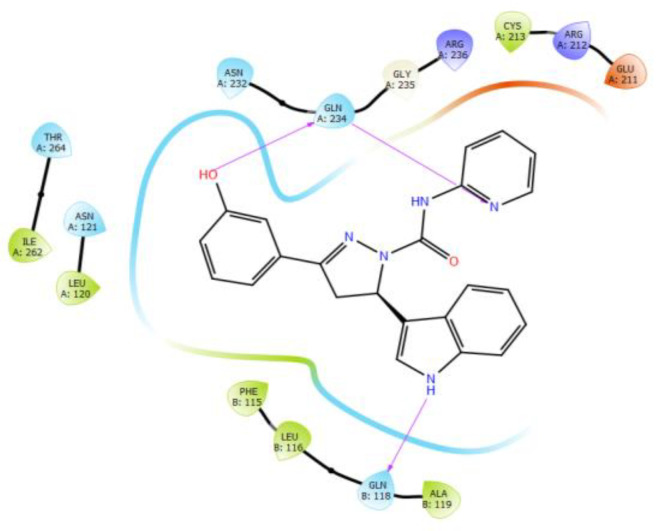
Two-dimensional representation of H-bonding interactions of compound after 4h with the binding site residues.

**Figure 6:**
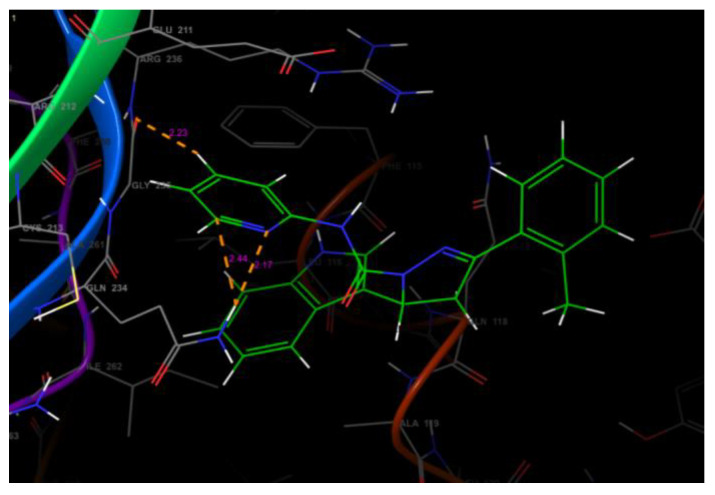
H-bonding interactions of compound after 4 d as visualized using pose viewer.

The receptor grid file was generated by choosing a random point in the binding site of choice by enclosing the grid box size in the workspace constrained to 8 Å. The molecules selected from LigPrep were subjected to flexible docking using the SP docking program set with default parameters. The docking score, the number of hydrogen bonds, amino acid residues, and the Glide score of the selected molecules along with the protein are summarized in Table 1 and Table 2. The compounds were found to possess a significant binding affinity with the binding site of the protein by interactions with the residues: Phe 115, Leu 116, Gln 118, Asn 121, Glu 211, Asn 232, Gln 234, Gln 235, Arg 236, Ile 262, and Thr 264, respectively. The 5-(indol-3-yl)-pyrazolines (4a–t) Glide G-score was in the range of −6.255 to −4.81, and the Glide energy range was from −43.506 to −36.142. The highest glide G-score was found to be −6.255, which corresponded to the compound 4h [3-(3-Hydroxyphenyl)-5-(1H-indol-3-yl)-N-(pyridin-2-yl)-4,5-dihydro-1H-pyrazole-1-carbox-amide], and was found to form three hydrogen bond interactions with the protein residues Leu 116 (1.95 Å, 1.72 Å) and Gln 118 (2.02 Å). The 2D and 3D representation of the binding pattern of the compounds are illustrated in Figure 2, Figure 3, Figure 4, Figure 5 and Figure 6. The H-bonding interactions formed by the ligands and protein residues are N–H⋯O, O–H⋯N and/or O–H···O. Apart from the H-bonding, other weaker interactions with the active site residues include electrostatic and hydrophobic bonds. Most of the compounds interacted via non-H-bonding to the Gln 171, Arg 212, Cys 213, Phe 214, Phe 233 residues with π-π stacking. The H-bond interactions of promising compounds along with the standard are presented in Table 2. The H-bond length and bonding interaction in compounds 4a-t are comparatively significant with respect to the standard. The basic nucleus of 5-(indol-3-yl)-4,5-dihydro-1H-pyrazole-1-carboxamide played an important role in the binding interactions as it possesses both the H-bond accepting and donating groups in the binding region of the protein, which allows the ligand to bind with high affinity and exhibit optimum binding conformation with high docking and the Glide G-score. In addition, the side chain of 2-amino pyridine and hydroxy substitution on the aryl ring in all the positions also contributed to a good binding score in case of all the molecules designed in this study. Based on the docking score and H-bonding interactions in the complex, the top 20 molecules were selected for synthesis and in vitro anti-proliferative activity evaluation.

### 3.2. Chemistry

In the last decade, an extensive number of chemically synthesized pyrazolines have been reported; however, only a limited number of them were synthesized using the relatively straightforward one-pot reaction method. In this study, we describe the synthesis method with good yield and reduced reaction time for the synthesis of selected new pyrazolines by employing a one-pot synthesis method, in turn eliminating any tedious purification steps for the removal of intermediate chalcones. The synthesis scheme of the designed pyrazolines is outlined in Figure 1. In this study, the pyrazolines (3a–k) were synthesized in a single reaction flask using a one-pot synthesis method utilizing indole-3-aldehyde with different substituted acetophenone and hydrazine [46]. 

The desired pyrazoline compounds 4a-t were produced successfully by treating pyrazolines (3a–k) with phenyl chloroformate in the presence of triethylamine in tetrahydrofuran at 25–30 °C. An equimolar quantity of triethylamine was used to neutralize the HCl fumes liberated during the reaction. No further isolation steps were necessary and the anhydride was yielded upon reaction with respective aryl/heteroaryl amines (4a–4t) [47].

The structures moieties of the newly synthesized compounds were confirmed with the help of their FT-IR, 1H NMR, mass spectroscopy, and elemental analysis (Supplementary Data). All the pyrazoline derivatives (4a–t) displayed characteristic N-H stretch (3288–3438 cm^−1^), C-H aromatic (3040–3134 cm^−1^), C-H aliphatic (2816–2979 cm^−1^), C=O (1662–1698 cm^−1^), C=N (1575–1630 cm^−1^), and C=C (1354–1607). The 1H NMR spectra of pyrazoline derivatives (4a–t) showed distinct and characteristic peaks. The protons were connected to the C4 and C5 carbon atoms of the 2-pyrazoline as a (ABX) spin system [48] which occurred as two doubles and one doublet (d) to doublet (dd) signals between δ 3.29–3.39, 3.72–3.92, and 6.05–6.32 ppm for three protons to prove a pyrazoline ring. The CH proton of the pyrrole ring in the indole appeared as a singlet (s) signal between δ 7.91–8.11 ppm, and NH-indole proton between δ 10.41–10.76 ppm also appeared as a singlet. Aryl protons exhibited its typical peaks between δ 6.49–8.03 ppm as multiplets, and Aryl/Het Aryl-NH protons δ 9.99–10.15 ppm as a singlet. The three protons of Ar-CH3, one proton of Ar-OH, and three protons of Ar-OCH3 appeared at δ 2.22, 9.75–9.97, and 3.64–3.82 ppm, respectively, as a singlet peak.

### 3.3. Anti-Proliferative Activity

The synthesized pure pyrazoline molecules were subjected to in vitro cytotoxicity evaluations in four human cancer cell lines which utilized the MTT assay, including breast (MCF-7), liver (Hep-2), lung (A-549), and prostate (DU-145). The indole derivative isatin was used as a standard compound for comparison for the tested cell lines. The IC_50_ values were determined for all the tested compounds, and are presented in Table 3. It was found that the majority of the tested molecules showed substantial anti-proliferative activity against the tested cell lines. The molecules 4g, 4, and 4q, in particular, showed high anticancer potential across all the cell lines used in this study. The compounds possessing o, m, p-hydroxy phenyl group substitution on the pyrazoline ring showed promising anticancer activities in all tested cell lines; however, this may also be attributed to the aminopyridine. As evident from the docking simulations, both aryl and pyridine rings play an important role in the H-bonding formation with the AhR–ARNT complex protein. 

The compounds containing hydroxy phenyl substitutions, such as 4g, 4q, and 4s, are relatively more potent among all the other tested compounds with IC_50_ values of 2.32 ± 0.11, 2.86 ± 0.12, and 3.02 ± 0.14 µM, respectively, when tested against the lung cancer cell line (A-549). Similarly, the compounds 4g, 4h, and 4q showed good anti-proliferative activity against the breast (MCF-7) cancer cell line with IC_50_ values of 43.59 ± 0.38, 47.45 ± 0.28, and 46.13 ± 0.34 µM, respectively. However, in case of the liver cancer cell line (Hep-2), the hydroxy phenyl substituted compounds showed IC_50_ values ranging between 21.12 ± 0.16 to 30.22 ± 0.19 µM, while other compounds containing unsubstituted phenyl (4a), and 2-Chloro phenyl substituted (4b) compounds also showed better anti-proliferative activity with IC_50_ values of 22.29 ± 0.30, 21.61 ± 0.25 µM, respectively. In the prostate cancer cell line (DU-145), the compounds with m-hydroxy phenyl substituted compounds (4h, 4n, 4r) with the irrespective pyridine group for carboxamide substitution showed promising inhibitory activity. In all the cases, some of the newly synthesized compounds showed promising activities than the standard compounds in the tested cell lines.

In most of the cases, hydroxy substitution at the 2-, 3- and 4-position of the phenyl ring led to substantial improvement in the therapeutic potency. Incorporation of a carboxamide group with various aminopyridines also resulted in a considerable improvement in anti-proliferative activity in most of the cell lines tested. Some of molecules synthesized in this study demonstrated notable therapeutic efficacy exclusively in the lung and prostate cancer cell line with IC_50_ values up to 2.32 ± 0.11 and 9.92 ± 0.13 µM, respectively. This enhanced anti-tumorigenic activity may be attributed to the inclusion of the indole moiety to the pyrazoline nucleus which may lead to possible interaction binding with the AhR–ARNT complex for enhanced chemotherapeutic effects. However, further in-depth investigations with suitable structural modifications in both the pyrazoline and indole moieties are required to gain a better understanding into the mechanism of action of indolyl dihydropyrazole derivatives.

## 4. Conclusions

This study describes the synthesis of a novel series of molecules designed in purview of the therapeutic properties of indole scaffold, incorporating a pyrazoline nucleus with the assumption of a possible interaction with an aryl hydrocarbon receptor for enhanced chemotherapeutic effects. The designed molecules were subjected to molecular docking analysis, and a few molecules were selected for synthesis and in vitro cytotoxicity testing using the MTT assay in different human cancer cell lines. Most of the synthesized compounds interacted with the AhR–ARNT complex by H-bonding interactions. The cytotoxicity assay of the hydroxy-phenyl-substituted compounds yielded potential anti-tumorigenic effects. The results of this study indicate that these scaffolds could be utilized as potential anticancer agents, and that the structural modification in both indole and pyrazoline nucleus play a major role in enhancing the anticancer efficiency.

## Figures and Tables

**Table 1:** Docking results of the selected compounds.

Compound	R	R1	Glide g-Score	Glide h-Bond	Glide Energy
4a	H	Pyridin-2-yl	−4.825	−0.25	−36.657
4b	2-Cl	Pyridin-2-yl	−4.901	−0.219	−37.331
4c	3-Cl	Pyridin-2-yl	−5.84	−0.268	−38.583
4d	3-CH3	Pyridin-2-yl	−5.071	−0.221	−37.89
4e	3-OCH3	Pyridin-2-yl	−4.892	−0.17	−38.54
4f	4-OCH3	Pyridin-2-yl	−4.982	−0.216	−38.981
4g	2-OH	Pyridin-2-yl	−5.835	−0.529	−39.868
4h	3-OH	Pyridin-2-yl	−6.255	−0.562	−38.825
4i	4-OH	Pyridin-2-yl	−4.81	−0.161	−36.75
4j	2-NO2	Pyridin-2-yl	−4.876	−0.237	−38.004
4k	3-NO2	Pyridin-2-yl	−4.939	−0.314	−39.227
4l	2-Cl	Pyridin-3-yl	−4.932	−0.157	−41.181
4m	2-OH	Pyridin-3-yl	−5.137	−0.329	−37.86
4n	3-OH	Pyridin-3-yl	−6.069	−0.449	−38.018
4o	4-Cl	Pyridin-4-yl	−4.882	−0.569	−41.003
4p	4-OCH3	Pyridin-4-yl	−4.816	−0.282	−36.142
4q	2-OH	Pyridin-4-yl	−5.022	−0.556	−41.616
4r	3-OH	Pyridin-4-yl	−6.152	−0.458	−38.088
4s	2-OH	Phenyl	−5.299	−0.32	−38.74
4t	4-OH	Phenyl	−6.17	−0.32	−43.506

**Table 2:** H-bonding interactions of compounds 4(a–t) with the protein residues.

Compound	R	R1	Amino Acid Residues (H-Bond Length Å)	No. of H-Bonding Interactions
4a	H	Pyridin-2-yl	Ile 262 (2.54)	1
4b	2-Cl	Pyridin-2-yl	Phe 115 (1.47)	1
4c	3-Cl	Pyridin-2-yl	Arg 236 (1.94), Phe 115 (1.06)	2
4d	3-CH3	Pyridin-2-yl	Ile 262 (2.05)	1
4e	3-OCH3	Pyridin-2-yl	Ile 262 (2.85), Phe 115 (3.02)	2
4f	4-OCH3	Pyridin-2-yl	Asn 121 (2.42)	1
4g	2-OH	Pyridin-2-yl	Ile 262 (1.65, 2.76), Gln 234 (2.12)	3
4h	3-OH	Pyridin-2-yl	Leu 116 (1.95, 1.72), Gln 118 (2.02)	3
4i	4-OH	Pyridin-2-yl	Gln 235 (3.00)	1
4j	2-NO2	Pyridin-2-yl	Arg 236 (2.99)	1
4k	3-NO2	Pyridin-2-yl	Arg 236 (3.13)	1
4l	2-Cl	Pyridin-3-yl	Leu 116 (2.20)	1
4m	2-OH	Pyridin-3-yl	Gln 118 (2.65)	1
4n	3-OH	Pyridin-3-yl	Asn 232 (3.25), Glu 211 (3.09), Arg 236 (2.04), Ile 262 (2.01)	4
4o	4-Cl	Pyridin-4-yl	Gln 234 (2.01), Ile 262 (3.33)	4
4p	4-OCH3	Pyridin-4-yl	Ile 262 (2.85), Phe 115 (3.02)	2
4q	2-OH	Pyridin-4-yl	Leu 116 (1.95, 1.72), Ile 262 (1.65, 2.76)	4
4r	3-OH	Pyridin-4-yl	Glu 211 (2.98), Arg 236 (2.62)	2
4s	2-OH	Phenyl	Gln 234 (3.44), Gln 118 (3.08), Thr 264 (2.89)	3
4t	4-OH	Phenyl	Gly 235 (2.93)	1

**Table 3:** Anticancer potential of compounds 4(a–t) in select experimental human cancer cell lines.

Compound	R	R1	IC_50_ (µM)			
			A-549	MCF-7	Hep G-2	DU-145
4a	H	Pyridin-2-yl	4.75 ± 0.21	54.32 ± 0.52	22.29 ± 0.30	19.53 ± 0.11
4b	2-Cl	Pyridin-2-yl	5.79 ± 0.13	59.01 ± 0.46	21.61 ± 0.25	19.68 ± 0.15
4c	3-Cl	Pyridin-2-yl	5.32 ± 0.17	60.64 ± 0.27	24.55 ± 0.26	16.54 ± 0.08
4d	3-CH3	Pyridin-2-yl	7.89 ± 0.26	56.58 ± 0.36	26.32 ± 0.14	16.02 ± 0.26
4e	3-OCH3	Pyridin-2-yl	5.78 ± 0.15	54.93 ± 0.31	32.57 ± 0.25	15.67 ± 0.21
4f	4-OCH3	Pyridin-2-yl	6.83 ± 0.16	58.76 ± 0.34	34.95 ± 0.13	16.84 ± 0.18
4g	2-OH	Pyridin-2-yl	2.32 ± 0.11	43.59 ± 0.38	24.53 ± 0.17	14.53 ± 0.16
4h	3-OH	Pyridin-2-yl	4.56 ± 0.14	47.45 ± 0.28	26.30 ± 0.14	12.36 ± 0.15
4i	4-OH	Pyridin-2-yl	5.74 ± 0.012	52.18 ± 0.12	30.22 ± 0.19	13.50 ± 0.14
4j	2-NO2	Pyridin-2-yl	7.77 ± 0.26	64.78 ± 0.46	35.50 ± 0.12	19.63 ± 0.15
4k	3-NO2	Pyridin-2-yl	8.81 ± 0.13	75.36 ± 0.34	33.77 ± 0.15	16.76 ± 0.17
4l	2-Cl	Pyridin-3-yl	6.46 ± 0.22	45.30 ± 0.26	27.74 ± 0.26	19.75 ± 0.12
4m	2-OH	Pyridin-3-yl	3.92 ± 0.24	57.18 ± 0.31	21.60 ± 0.29	10.15 ± 0.13
4n	3-OH	Pyridin-3-yl	5.45 ± 0.14	55.74 ± 0.42	29.52 ± 0.21	13.99 ± 0.19
4o	4-Cl	Pyridin-4-yl	6.78 ± 0.16	54.84 ± 0.27	30.53 ± 0.19	14.65 ± 0.21
4p	4-OCH3	Pyridin-4-yl	5.74 ± 0.15	54.23 ± 0.29	26.25 ± 0.24	15.52 ± 0.15
4q	2-OH	Pyridin-4-yl	2.86 ± 0.12	46.13 ± 0.34	21.12 ± 0.16	9.92 ± 0.13
4r	3-OH	Pyridin-4-yl	4.50 ± 0.18	56.50 ± 0.37	25.87 ± 0.15	12.16 ± 0.17
4s	2-OH	Phenyl	3.02 ± 0.14	48.67 ± 0.34	22.28 ± 0.17	10.47 ± 0.16
4t	4-OH	Phenyl	5.26 ± 0.19	66.53 ± 0.31	25.94 ± 0.12	12.19 ± 0.22
Isatin	--		4.96 ± 0.21	32.61 ± 0.25	28.29 ± 0.14	19.64 ± 0.12

## Supplementary Materials

Supplementary Data are available online.

## Sample Availability

Samples of the compounds are available from the authors.
