# Supplementary material for: Design, Synthesis, and In Vitro Evaluation of Novel Indolyl DiHydropyrazole Derivatives as Potential Anticancer Agents
Source: Molecules. 2021 Aug 29;26(17):5235. doi: 10.3390/molecules26175235 (PMC8434462; doi:10.3390/molecules26175235)
Supplement: Supplementary file 1 [file molecules-26-05235-s001.zip › molecules-1254174-supplementary.pdf]

molecules
MDPI

Design, Synthesis, and In vitro Evaluation of Novel Indolyl Di-Hydropyrazole Derivatives as Potential Anticancer Agents

Katharigatta N. Venugopala [1,2,*], Mohammed Habeebuddin [3], Bandar E. Aldhubiab [1] and Afzal Haq Asif [4,*]

[1] Department of Pharmaceutical Sciences, College of Clinical Pharmacy, King Faisal University, Al-Ahsa 31982, Saudi Arabia; baldhubiab@kfu.edu.sa
[2] Department of Biotechnology and Food Technology, Durban University of Technology, Durban 4001, South Africa
[3] Department of biomedical sciences, College of medicine, King Faisal University, Al-Ahsa 31982, Saudi Arabia; Hmohammed@kfu.edu.sa
[4] Department of Pharmacy Practice, College of Clinical Pharmacy, King Faisal University, Al-Ahsa 31982, Saudi Arabia
* Correspondence: kvenugopala@kfu.edu.sa (K.N.V.); ahasif@kfu.edu.sa (A.H.A)

Supplementary Data:

Structural & Molecular Docking Data of the Designing Compounds:

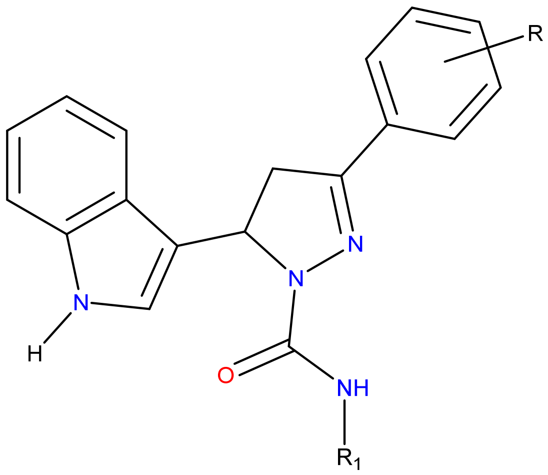

Title	R	R_1	Docking score	Glide gscore	Glide hbond	Glide energy	New code
1	2-Me	2-Pyridinyl	−4.508	−4.508	−0.265	−35.167	
2	3-Me	2-Pyridinyl	−5.071	−5.071	−0.221	−37.89	4d
3	4-Me	2-Pyridinyl	−3.965	−3.965	−0.228	−38.321	
4	2-Cl	2-Pyridinyl	−4.901	−4.901	−0.219	−37.331	4b
5	3-Cl	2-Pyridinyl	−5.84	−5.84	−0.268	−38.583	4c
6	4-Cl	2-Pyridinyl	−3.995	−3.995	−0.228	−39.915	
7	2-OH	2-Pyridinyl	−5.835	−5.835	−0.529	−39.868	4g
8	3-OH	2-Pyridinyl	−6.255	−6.255	−0.562	−38.825	4h
9	4-OH	2-Pyridinyl	−4.81	−4.81	−0.161	−36.75	4i
10	2-OCH_3	2-Pyridinyl	−4.357	−4.357	0	−35.931	
11	3-OCH_3	2-Pyridinyl	−4.892	−4.892	−0.17	−38.54	4e
12	4-OCH_3	2-Pyridinyl	−4.982	−4.982	−0.216	−38.981	4f
13	2-NO_2	2-Pyridinyl	−4.876	−4.876	−0.237	−38.004	4j
14	3-NO_2	2-Pyridinyl	−4.939	−4.939	−0.314	−39.227	4k
15	4-NO_2	2-Pyridinyl	−4.491	−4.491	−0.324	−35.921	
16	H	2-Pyridinyl	−4.825	−4.825	−0.25	−36.657	4a
17	2-Me	3- Pyridinyl	−4.127	−4.127	0	−35.924	

18	3-Me	3- Pyridinyl	−4.158	−4.158	−0.032	−36.743	
19	4-Me	3- Pyridinyl	−5.296	−5.296	0	−38.464	
20	3-Cl	3- Pyridinyl	−3.854	−3.854	−0.087	−32.076	
21	2-Cl	3- Pyridinyl	−4.932	−4.932	−0.157	−41.181	4l
22	4-Cl	3- Pyridinyl	−4.237	−4.237	−0.245	−39.097	
23	2-OH	3- Pyridinyl	−5.137	−5.137	−0.329	−37.86	4m
24	3-OH	3- Pyridinyl	−6.069	−6.069	−0.449	−38.018	4n
25	4-OH	3- Pyridinyl	−4.755	−4.755	0	−38.462	
26	2-OCH_3	3- Pyridinyl	−4.445	−4.445	0	−37.192	
27	3-OCH_3	3- Pyridinyl	−4.377	−4.377	0	−36.252	
28	2-OCH_3	3- Pyridinyl	−4.471	−4.471	−0.32	−38.683	
29	2-NO_2	3- Pyridinyl	−3.965	−3.965	0	−34.243	
30	3-NO_2	3- Pyridinyl	−3.864	−3.864	−0.037	−34.941	
31	4-NO_2	3- Pyridinyl	−3.18	−3.18	0	−35.521	
32	H	3- Pyridinyl	−4.472	−4.472	0	−35.94	
33	2-Me	4- Pyridinyl	−4.612	−4.612	−0.154	−38.57	
34	3-Me	4- Pyridinyl	−4.416	−4.416	−0.353	−39.095	
35	4-Me	4- Pyridinyl	−4.138	−4.138	−0.152	−34.895	
36	2-Cl	4- Pyridinyl	−4.174	−4.174	−0.152	−38.637	
37	3-Cl	4- Pyridinyl	−4.131	−4.131	0	−37.215	
38	4-Cl	4- Pyridinyl	−4.882	−4.882	−0.569	−41.003	4o
39	2-OH	4- Pyridinyl	−5.022	−5.022	−0.556	−41.616	4q
40	3-OH	4- Pyridinyl	−6.152	−6.152	−0.458	−38.088	4r
41	4-OH	4- Pyridinyl	−4.558	−4.558	−0.231	−35.527	
42	2-OCH_3	4- Pyridinyl	−4.438	−4.438	−0.32	−31.995	
43	3-OCH_3	4- Pyridinyl	−4.384	−4.384	0	−38.514	
44	4-OCH_3	4- Pyridinyl	−4.816	−4.816	−0.282	−36.142	4p
45	2-NO_2	4- Pyridinyl	−4.298	−4.298	0	−35.35	
46	3-NO_2	4- Pyridinyl	−4.238	−4.238	−0.16	−39.242	
47	4-NO_2	4- Pyridinyl	−4.147	−4.147	−0.348	−38.333	
48	H	4- Pyridinyl	−4.385	−4.385	−0.16	−38.107	
49	2-Me	Phenyl	−4.387	−4.387	0	−34.063	
50	3-Me	Phenyl	−4.517	−4.517	0	−36.077	
51	4-Me	Phenyl	−5.286	−5.286	0	−38.361	
52	2-Cl	Phenyl	−4.335	−4.335	0	−36.068	
53	3-Cl	Phenyl	−4.499	−4.499	0	−36.461	
54	4-Cl	Phenyl	−3.515	−3.515	−0.049	−32.591	
55	2-OH	Phenyl	−5.299	−5.299	−0.32	−38.74	4s
56	3-OH	Phenyl	−4.693	−4.693	−0.17	−40.779	
57	4-OH	Phenyl	−6.17	−6.17	−0.32	−43.506	4t
58	2-OCH_3	Phenyl	−4.827	−4.827	0	−36.287	
59	3-OCH_3	Phenyl	−4.538	−4.538	−0.16	−39.389	
60	4-OCH_3	Phenyl	−4.376	−4.376	−0.32	−37.939	
61	2-NO_2	Phenyl	−4.34	−4.34	0	−35.005	
62	3-NO_2	Phenyl	−4.373	−4.373	0	−39.133	
63	4-NO_2	Phenyl	−5.804	−5.804	0	−46.52	
64	H	Phenyl	−4.449	−4.449	0	−35.581	

Spectral Data of the Synthesized compounds:

3-Phenyl-5-(1H-indol-3-yl)-N-(pyridin-2-yl)-4,5-dihydro-1H-pyrazole-1-carboxamide(4a*):* Pale yellow crystals, MF: $C_{23}H_{19}N_5O$; MW: 381; MP = 122–124 °C, % Yield: 56; R_f = 0.45 (4:6, Pet. ether: Ethyl acetate). Anal. Calcd for $C_{23}H_{19}N_5O$ (381): C, 72.42; H, 5.02; N, 18.36. Found: C, 72.68; H, 5.01; N, 18.40. IR (cm^{-1}): 3438 (NH), 3050–3094 (C-H aromatic), 2924–2878 (C-H aliphatic), 1694 (C=O), 1612 (C=N), 1406-1576 (C=C). ^{1}HNMR (DMSO-d_6): δ 3.34 (d, *J*=7.0 Hz, 1H, Pyrazoline H_a), 3.87 (d, *J*=7.0 Hz,1H, Pyrazoline H_b), 6.32 (dd, *J*=7.5 Hz, 1H, Pyrazoline H_c), 6.72–7.87 (m, *J*=7.5 Hz, 13H, Ar-H), 7.99 (s, 1H, CH-pyrrole), 10.07 (s, 1H, NH-Ar), 10.61 (s, 1H, NH-Indole). FAB-MS (*m*/*z*): 382 $[M+1]^+$.

3-(2-Chlorophenyl)-5-(1H-indol-3-yl)-N-(pyridin-2-yl)-4,5-dihydro-1H-pyrazole-1-carboxamide (4b*):* Pale yellow crystals, MF: $C_{23}H_{18}ClN_5O$; MW: 415; MP = 178–180 °C; % Yield: 48; R_f = 0.42 (4:6, Pet. ether: Ethyl acetate). Anal. Calcd for $C_{23}H_{18}ClN_5O$ (415): C, 66.43; H, 4.36; N, 16.84. Found: C, 66.36; H, 4.37; N, 16.87. IR (cm^{-1}): 3355 (NH), 3057–3016 (C-H aromatic), 2912–2954 (C-H aliphatic), 1666 (C=O), 1604 (C=N), 1456-1597 (C=C). ^{1}HNMR (DMSO-d_6): δ 3.33 (d, *J*=7.0 Hz, 1H, Pyrazoline H_a), 3.77 (d, *J*=7.0 Hz, 1H, Pyrazoline H_b), 6.28 (dd, *J*=7.5 Hz, 1H, Pyrazoline H_c), 6.83–7.84 (m, *J*=7.5 Hz, 12H, Ar-H), 7.96 (s, 1H, CH-pyrrole), 10.11 (s, 1H, NH-Ar), 10.59 (s, 1H, NH-Indole). FAB-MS (*m*/*z*): 417 $[M+2]^+$.

3-(3-Chlorophenyl)-5-(1H-indol-3-yl)-N-(pyridin-2-yl)-4,5-dihydro-1H-pyrazole-1-carboxamide (4c*):* Pale yellow crystals, MF: $C_{23}H_{18}ClN_5O$; MW: 415; MP = 170–172 °C; % Yield: 85; R_f = 0.42 (4:6, Pet. ether: Ethyl acetate). Anal. Calcd for $C_{23}H_{18}ClN_5O$ (415): C, 66.43; H, 4.36; N, 16.84. Found: C, 66.42; H, 4.35; N, 16.85. IR (cm^{-1}): 3354 (NH), 3060–3104 (C-H aromatic), 2930–2860 (C-H aliphatic), 1692 (C=O), 1596 (C=N), 1467-1511 (C=C). ^{1}HNMR (DMSO-d_6): δ 3.35 (d, *J*=7.0 Hz, 1H, Pyrazoline H_a), 3.78 (d, *J*=7.0 Hz, 1H, Pyrazoline H_b), 6.07 (dd, *J*=7.5 Hz, 1H, Pyrazoline H_c), 6.84–7.86 (m, *J*=7.5 Hz, 12H, Ar-H), 7.96 (s, 1H, CH-pyrrole), 10.12 (s, 1H, NH-Ar), 10.47 (s, 1H, NH-Indole). FAB-MS (*m*/*z*): 417 $[M+2]^+$.

3-(3-Methylphenyl)-5-(1H-indol-3-yl)-N-(pyridin-2-yl)-4,5-dihydro-1H-pyrazole-1-carboxamide (4d*):* Pale yellow crystals, MF: $C_{24}H_{21}N_5O$; MW: 395; MP = 186–188 °C; % Yield: 67; R_f = 0.40 (4:6, Pet. ether: Ethyl acetate). Anal. Calcd $C_{24}H_{21}N_5O$ (395): C, 72.89; H, 5.35; N, 17.71. Found: C, 73.04; H, 5.36; N, 17.75. IR (cm^{-1}): 3436 (NH), 3057-3119 (C-H aromatic), 2931-2904 (C-H aliphatic), 1695 (C=O), 1596 (C=N), 1487-1567 (C=C). ^{1}HNMR (DMSO-d_6): δ 2.22 (s, 3H, Ar-CH_3), 3.36 (d, *J*=7.0 Hz, 1H, Pyrazoline H_a), 3.72 (d, *J*=7.0 Hz, 1H, Pyrazoline H_b), 6.32 (dd, *J*=7.5 Hz, 1H, Pyrazoline H_c), 6.83–7.86 (m, *J*=7.5 Hz, 12H, Ar-H), 7.92 (s, 1H, CH-pyrrole), 10.09 (s, 1H, NH-Ar), 10.55 (s, 1H, NH-Indole). FAB-MS (*m*/*z*): 396 $[M+1]^+$.

3-(3-Methoxyphenyl)-5-(1H-indol-3-yl)-N-(pyridin-2-yl)-4,5-dihydro-1H-pyrazole-1-carbox-amide (4e*):* Pale yellow crystals, MF: $C_{24}H_{21}N_5O_2$; MW: 411; MP = 162–164 °C; % Yield: 48; R_f = 0.38 (4:6, Pet. ether: Ethyl acetate). Anal. Calcd for $C_{24}H_{21}N_5O_2$ (411): C, 70.06; H, 5.14; N, 17.02. Found: C, 70.16; H, 5.13; N, 17.05. IR (cm^{-1}): 3357 (NH), 3056-3108 (C-H

aromatic), 2975-2931 (C-H aliphatic), 1670 (C=O), 1597 (C=N), 1415-1544 (C=C). ^{1}HNMR (DMSO-d_6): δ 3.34 (d, *J*=7.0 Hz, 1H, Pyrazoline Ha), 3.68 (s, 3H, Ar-OCH_3), 3.78 (d, *J*=7.0 Hz, 1H, Pyrazoline Hb), 6.31 (dd, *J*=7.5 Hz, 1H, Pyrazoline Hc), 6.60–7.83 (m, *J*=7.5 Hz, 12H, Ar-H), 8.01 (s, 1H, CH-pyrrole), 10.14 (s, 1H, NH-Ar), 10.63 (s, 1H, NH-Indole). FAB-MS (*m*/*z*): 412 $[M+1]^+$.

3-(4-Methoxyphenyl)-5-(1H-indol-3-yl)-N-(pyridin-2-yl)-4,5-dihydro-1H-pyrazole-1-carbox-amide (4f*):* Pale yellow crystals, MF: $C_{24}H_{21}N_5O_2$; MW: 411; MP = 196–198 °C; % Yield: 92; R_f = 0.38 (4:6, Pet. ether: Ethyl acetate). Anal. Calcd for $C_{24}H_{21}N_5O_2$ (411): C, 70.06; H, 5.14; N, 17.02. Found: C, 69.96; H, 5.13; N, 16.94. IR (cm^{-1}): 3356 (NH), 3052–3106 (C-H aromatic), 2927–2904 (C-H aliphatic), 1698 (C=O), 1593 (C=N), 1442–1573 (C=C). ^{1}HNMR (DMSO-d_6): δ 3.29 (d, *J*=7.0 Hz, 1H, Pyrazoline H_a), 3.64 (s, 3H, Ar-OCH_3), 3.89 (d, *J*=7.0 Hz, 1H, Pyrazoline H_b), 6.23 (dd, *J*=7.5 Hz, 1H, Pyrazoline H_c), 6.74–7.86 (m, *J*=7.5 Hz, 12H, Ar-H), 8.04 (s, 1H, CH-pyrrole), 10.01 (s, 1H, NH-Ar), 10.58 (s, 1H, NH-Indole). FAB-MS (*m*/*z*): 412 $[M+1]^+$.

3-(2-Hydroxyphenyl)-5-(1H-indol-3-yl)-N-(pyridin-2-yl)-4,5-dihydro-1H-pyrazole-1-carbox-amide (4g*):* Pale yellow crystals, MF: $C_{23}H_{19}N_5O_2$; MW: 397; MP = 140–142 °C; % Yield: 46; R_f= 0.35 (4:6, Pet. ether: Ethyl acetate). Anal. Calcd for $C_{23}H_{19}N_5O_2$ (397): C, 69.51; H, 4.82; N, 17.62. Found: C, 69.70; H, 4.84; N, 17.66. IR (cm^{-1}): 3353 (NH), 3063–3108 (C-H aromatic), 2960–2816 (C-H aliphatic), 1688 (C=O), 1609 (C=N), 1560-1584 (C=C). ^{1}HNMR (DMSO-d_6): δ 3.38 (d, *J*=7.0 Hz, 1H, Pyrazoline Ha), 3.86 (d, *J*=7.0 Hz, 1H, Pyrazoline Hb), 6.10 (dd, *J*=7.5 Hz, 1H, Pyrazoline Hc), 6.49–7.85 (m, *J*=7.5 Hz, 12H, Ar-H), 7.97 (s, 1H, CH-pyrrole), 9.80 (s, 1H, Ar-OH), 9.99 (s, 1H, NH-Ar), 10.60 (s, 1H, NH-Indole). FAB-MS (*m*/*z*): 398 $[M+1]^+$.

3-(3-Hydroxyphenyl)-5-(1H-indol-3-yl)-N-(pyridin-2-yl)-4,5-dihydro-1H-pyrazole-1-carbox-amide (4h*):* Pale yellow crystals, MF: $C_{23}H_{19}N_5O_2$; MW: 397; MP = 164–166 °C; % Yield: 50; R_f= 0.35 (4:6, Pet. ether: Ethyl acetate). Anal. Calcd for $C_{23}H_{19}N_5O_2$ (397): C, 69.51; H, 4.82; N, 17.62. Found: C, 69.72; H, 4.84; N, 17.89. IR (cm^{-1}): 3288 (NH), 3064–3101 (C-H aromatic), 2920-2850 (C-H aliphatic), 1674 (C=O), 1594 (C=N), 1439-1546 (C=C). ^{1}HNMR (DMSO-d_6): δ 3.36 (d, *J*=7.0 Hz, 1H, Pyrazoline H_a), 3.92 (d, *J*=7.0 Hz, 1H, Pyrazoline H_b), 6.32 (dd, *J*=7.5 Hz, 1H, Pyrazoline H_c), 6.63–8.03 (m, *J*=7.5 Hz, 12H, Ar-H), 8.11 (s, 1H, CH-pyrrole), 9.79 (s, 1H, Ar-OH), 10.09 (s, 1H, NH-Ar), 10.59 (s, 1H, NH-Indole). FAB-MS (*m*/*z*): 398 $[M+1]^+$.

3-(4-Hydroxyphenyl)-5-(1H-indol-3-yl)-N-(pyridin-2-yl)-4,5-dihydro-1H-pyrazole-1-carbox- amide (4i*):* Pale yellow crystals, MF: $C_{23}H_{19}N_5O_2$; MW: 397; MP = 140–142 °C; % Yield: 42; R_f = 0.35 (4:6, Pet. ether: Ethyl acetate). Anal. Calcd for $C_{23}H_{19}N_5O_2$ (397): C, 69.51; H, 4.82; N, 17.62. Found: C, 69.94; H, 4.85; N, 17.63. IR (cm^{-1}): 3350 (NH), 3073–3124 (C-H

aromatic), 2965–2923 (C-H aliphatic), 1672 (C=O), 1575 (C=N), 1398–1489 (C=C). ^{1}HNMR (DMSO-d_6): δ 3.33(d, *J*=7.0 Hz, 1H, Pyrazoline H$_a$), 3.87 (d, *J*=7.0 Hz, 1H, Pyrazoline H$_b$), 6.21 (dd, *J*=7.5 Hz, 1H, Pyrazoline H$_c$), 6.83–7.83 (m, *J*=7.5 Hz, 12H, Ar-H), 7.91 (s, 1H, CH-pyrrole), 9.75 (s, 1H, Ar-OH), 10.15(s, 1H, NH-Ar), 10.49 (s, 1H, NH-Indole). FAB-MS (*m*/*z*): 398 [M+1]$^+$.

3-(2-Nitrophenyl)-5-(1H-indol-3-yl)- N-(pyridin-2-yl)-4,5-dihydro-1H-pyrazole-1-carboxamide (4j)*:* Pale yellow crystals, MF: $C_{23}H_{18}N_6O_3$; MW: 426; MP = 158–160 °C, % Yield: 44; R$_f$= 0.42 (4:6, Pet. ether: Ethyl acetate). Anal. Calcd for $C_{23}H_{18}N_6O_3$ (426): C, 64.78; H, 4.25; N, 19.71. Found: C, 64.82; H, 4.26; N, 19.67. IR (cm^{-1}): 3401 (NH), 3075–3126 (C-H aromatic), 2931–2862 (C-H aliphatic), 1676 (C=O), 1602 (C=N), 1545-1588 (C=C). ^{1}HNMR (DMSO-d_6): δ 3.37 (d, *J*=7.0 Hz, 1H, Pyrazoline H$_a$), 3.87 (d, *J*=7.0 Hz, 1H, Pyrazoline H$_b$), 6.31 (dd, *J*=7.5 Hz, 1H, Pyrazoline H$_c$), 6.85–7.87 (m, *J*=7.5 Hz, 12H, Ar-H), 8.01(s, 1H, CH-pyrrole), 10.10 (s, 1H, NH-Ar), 10.68 (s, 1H, NH-Indole). FAB-MS (*m*/*z*): 427 [M+1]$^+$.

3-(3-Nitrophenyl)-5-(1H-indol-3-yl)-N-(pyridin-2-yl)-4,5-dihydro-1H-pyrazole-1-carboxamide (4k)*:* Pale yellow crystals, MF:$C_{23}H_{18}N_6O_3$; MW: 426; MP = 150–152 °C; % Yield: 53; R$_f$= 0.42 (4:6, Pet. ether: Ethyl acetate). Anal. Calcd for $C_{23}H_{18}N_6O_3$ (426): C, 64.78; H, 4.25; N, 19.71. Found: C, 64.88; H, 4.27; N, 19.70. IR (cm^{-1}): 3406 (NH), 3087–3134 (C-H aromatic), 2920–2846 (C-H aliphatic), 1668 (C=O), 1626 (C=N), 1493–1597 (C=C). ^{1}HNMR (DMSO-d_6): δ 3.34 (d, *J*=7.0 Hz, 1H, Pyrazoline H$_a$), 3.87 (d, *J*=7.0 Hz, 1H, Pyrazoline H$_b$), 6.27 (dd, *J*=7.5 Hz, 1H, Pyrazoline H$_c$), 6.84–7.86 (m, *J*=7.5 Hz, 12H, Ar-H), 7.98 (s, 1H, CH-pyrrole), 10.07 (s, 1H, NH-Ar), 10.76 (s, 1H, NH-Indole). FAB-MS (*m*/*z*): 427 [M+1]$^+$.

3-(3-Chlorophenyl)-5-(1H-indol-3-yl)-N-(pyridin-3-yl)-4,5-dihydro-1H-pyrazole-1-carboxamide (4l)*:* Pale yellow crystals, MF: $C_{23}H_{18}ClN_5O$; MW: 415; MP = 124–126 °C; % Yield: 86; R$_f$= 0.42 (4:6, Pet. ether: Ethyl acetate). Anal. Calcd for $C_{23}H_{18}ClN_5O$ (415): C, 72.89; H, 5.35; N, 17.71. Found: C, 72.84; H, 5.34; N, 17.72. IR (cm^{-1}): 3396 (NH), 3052–3098 (C-H aromatic), 2963–2878 (C-H aliphatic), 1662 (C=O), 1600 (C=N), 1460–1515 (C=C). ^{1}HNMR (DMSO-d_6): δ 3.33 (d, *J*=7.0 Hz, 1H, Pyrazoline H$_a$), 3.77 (d, *J*=7.0 Hz, 1H, Pyrazoline H$_b$), 6.32 (dd, *J*=7.5 Hz, 1H, Pyrazoline H$_c$), 6.83–7.84 (m, *J*=7.5 Hz, 12H, Ar-H), 7.93 (s, 1H, CH-pyrrole), 10.10 (s, 1H, NH-Ar), 10.62 (s, 1H, NH-Indole). FAB-MS (*m*/*z*): 417 [M+2]$^+$.

3-(2-Hydroxyphenyl)-5-(1H-indol-3-yl)-N-(pyridin-3-yl)-4,5-dihydro-1H-pyrazole-1-carbox-amide (4m)*:* Pale yellow crystals, MF: $C_{23}H_{19}N_5O_2$; MW: 397; MP = 128–130 °C, % Yield: 64; R$_f$= 0.35 (4:6, Pet. ether: Ethyl acetate). Anal. Calcd for $C_{23}H_{19}N_5O_2$ (397): C, 69.51; H, 4.82; N, 17.62. Found: C, 69.72; H, 4.81; N, 17.64. IR (cm^{-1}): 3420 (NH), 3086–3133 (C-H aromatic), 2916–2979 (C-H aliphatic), 1681 (C=O), 1599 (C=N), 1396–1540 (C=C). ^{1}HNMR (DMSO-d_6): δ 3.39 (d, *J*=7.0 Hz, 1H, Pyrazoline H$_a$), 3.78 (d, *J*=7.0 Hz, 1H, Pyrazoline H$_b$), 6.18 (dd, *J*=7.5 Hz, 1H, Pyrazoline H$_c$), 6.53–7.86 (m, *J*=7.5 Hz, 12H, Ar-H), 7.92 (s, 1H, CH-pyrrole), 9.89 (s, 1H, Ar-OH), 10.08 (s, 1H, NH-Ar), 10.47 (s, 1H, NH-Indole). FAB-MS (*m*/*z*):

398 [M+1]+.

3-(3-Hydroxyphenyl)-5-(1H-indol-3-yl)-N-(pyridin-3-yl)-4,5-dihydro-1H-pyrazole-1-carbox-amide (4n)*:* Pale yellow crystals, MF: $C_{23}H_{19}N_5O_2$; MW: 397; MP = 110–112 °C; % Yield: 78; R_f= 0.35 (4:6, Pet. ether: Ethyl acetate). Anal. Calcd for $C_{23}H_{19}N_5O_2$ (397): C, 69.51; H, 4.82; N, 17.62. Found: C, 69.54; H, 4.80; N, 17.58. IR (cm^{-1}): 3424 (NH), 3074–3112 (C-H aromatic), 2920–2850 (C-H aliphatic), 1696 (C=O), 1598 (C=N), 1462-1546 (C=C). ^{1}HNMR (DMSO-d_6): δ 3.36 (d, *J*=7.0 Hz, 1H, Pyrazoline H_a), 3.87 (d, *J*=7.0 Hz, 1H, Pyrazoline H_b), 6.09 (dd, *J*=7.5 Hz, 1H, Pyrazoline H_c), 6.72–7.86 (m, *J*=7.5 Hz, 12H, Ar-H), 7.93 (s, 1H, CH-pyrrole), 9.90 (s, 1H, Ar-OH), 10.08 (s, 1H, NH-Ar), 10.64 (s, 1H, NH-Indole). FAB-MS (*m*/*z*): 398 [M+1]+.

3-(4-Chlorophenyl)-5-(1H-indol-3-yl)-N-(pyridin-4-yl)-4,5-dihydro-1H-pyrazole-1-carboxamide (**4o**)*:* Pale yellow crystals, MF: $C_{23}H_{18}ClN_5O$; MW: 415; MP = 168–170 °C, % Yield: 48; R_f= 0.42 (4:6, Pet. ether: Ethyl acetate). Anal. Calcd for $C_{23}H_{18}ClN_5O$ (415): C, 72.89; H, 5.35; N, 17.71. Found: C, 73.04; H, 5.36; N, 17.68. IR (cm^{-1}): 3400 (NH), 3067–3106 (C-H aromatic), 2973–2835 (C-H aliphatic), 1682 (C=O), 1607 (C=N), 1494-1599 (C=C). ^{1}HNMR (DMSO-d_6): δ 3.34 (d, *J*=7.0 Hz, 1H, Pyrazoline H_a), 3.78 (d, *J*=7.0 Hz, 1H, Pyrazoline H_b), 6.31 (dd, *J*=7.5 Hz, 1H, Pyrazoline H_c), 6.56–7.85 (m, *J*=7.5 Hz, 12H, Ar-H), 7.96 (s, 1H, CH-pyrrole), 10.09 (s, 1H, NH-Ar), 10.42 (s, 1H, NH-Indole). FAB-MS (*m*/*z*): 417 [M+2]+

3-(4-Methoxyphenyl)-5-(1H-indol-3-yl)-N-(pyridin-4-yl)-4,5-dihydro-1H-pyrazole-1-carbox-amide (4p)*:* Pale yellow crystals, MF: $C_{24}H_{21}N_5O_2$; MW: 411; MP = 140-142 °C, % Yield: 56; R_f= 0.38 (4:6, Pet. ether: Ethyl acetate). Anal. Calcd for $C_{24}H_{21}N_5O_2$ (411): C, 70.06; H, 5.14; N, 17.02. Found: C, 70.12; H, 5.16; N, 16.99. IR (cm^{-1}): 3409 (NH), 3076–3022 (C-H aromatic), 2927–2854 (C-H aliphatic), 1684 (C=O), 1595 (C=N), 1412-1568 (C=C). ^{1}HNMR (DMSO-d_6): δ 3.38 (d, *J*=7.0 Hz, 1H, Pyrazoline H_a), 3.82 (s, 3H, Ar-OCH_3), 3.87 (d, *J*=7.0 Hz, 1H, Pyrazoline H_b), 6.07 (dd, *J*=7.5 Hz, 1H, Pyrazoline H_c), 6.84–7.87 (m, *J*=7.5 Hz, 12H, Ar-H), 7.94 (s, 1H, CH-pyrrole), 10.15 (s, 1H, NH-Ar), 10.69 (s, 1H, NH-Indole). FAB-MS (*m*/*z*): 412 [M+1]+.

3-(2-Hydroxyphenyl)-5-(1H-indol-3-yl)-N-(pyridin-4-yl)-4,5-dihydro-1H-pyrazole-1-carbox-amide (4q)*:* Pale yellow crystals, MF: $C_{23}H_{19}N_5O_2$; MW: 397; MP = 188–190 °C; % Yield: 64; R_f= 0.35 (4:6, Pet. ether: Ethyl acetate). Anal. Calcd for $C_{23}H_{19}N_5O_2$ (397): C, 69.51; H, 4.82; N, 17.62. Found: C, 69.44; H, 4.83; N, 17.56. IR (cm^{-1}): 3388 (NH), 3057–3120 (C-H aromatic), 2967–2835 (C-H aliphatic), 1678 (C=O), 1605 (C=N), 1354-1590 (C=C). ^{1}HNMR (DMSO-d_6): δ 3.37 (d, *J*=7.0 Hz, 1H, Pyrazoline H_a), 3.79 (d, *J*=7.0 Hz, 1H, Pyrazoline H_b), 6.08 (dd, *J*=7.5 Hz, 1H, Pyrazoline H_c), 6.35–8.04 (m, *J*=7.5 Hz, 12H, Ar-H), 8.10 (s, 1H, CH-pyrrole), 9.97 (s, 1H, Ar-OH), 10.06 (s, 1H, NH-Ar), 10.55 (s, 1H, NH-Indole). FAB-MS (*m*/*z*): 398 [M+1]+.

3-(3-Hydroxyphenyl)-5-(1H-indol-3-yl)-N-(pyridin-4-yl)-4,5-dihydro-1H-pyrazole-1-carbox-amide (4r): Pale yellow crystals, MF: $C_{23}H_{19}N_5O_2$; MW: 397; MP = 174–176 °C, % Yield: 52; R_f = 0.35 (4:6, Pet. ether: Ethyl acetate). Anal. Calcd for $C_{23}H_{19}N_5O_2$ (397): C, 69.51; H, 4.82; N, 17.62. Found: C, 69.44; H, 4.82; N, 17.60. IR (cm^{-1}): 3386 (NH), 3072–3101 (C-H aromatic), 2931-2848 (C-H aliphatic), 1679 (C=O), 1630 (C=N), 1464-1511 (C=C). ^{1}HNMR (DMSO-d_6): δ 3.38 (d, *J*=7.0 Hz, 1H, Pyrazoline H_a), 3.73 (d, *J*=7.0 Hz, 1H, Pyrazoline H_b), 6.28 (dd, *J*=7.5 Hz, 1H, Pyrazoline H_c), 6.72–7.87 (m, *J*=7.5 Hz, 12H, Ar-H), 8.00 (s, 1H, CH-pyrrole), 9.89 (s, 1H, Ar-OH), 10.13 (s, 1H, NH-Ar), 10.41 (s, 1H, NH-Indole). FAB-MS (*m/z*): 398 $[M+1]^+$.

3-(2-Hydroxyphenyl)-5-(1H-indol-3-yl)-N-phenyl-4,5-dihydro-1H-pyrazole-1-carboxamide (4s): Pale yellow crystals, MF: $C_{24}H_{20}N_4O_2$; MW: 396; MP = 57 °C; % Yield:132-134; R_f = 0.35 (4:6, Pet. ether: Ethyl acetate). Anal. Calcd for $C_{24}H_{20}N_4O_2$ (396): C, 72.71; H, 5.08; N, 14.13. Found: C, 72.96; H, 5.07; N, 14.10. IR (cm^{-1}): 3404 (NH), 3040-3110 (C-H aromatic), 2924-2854 (C-H aliphatic), 1680 (C=O), 1628 (C=N), 1465-1607 (C=C). ^{1}HNMR (DMSO-d_6): δ 3.34 (d, *J*=7.0 Hz, 1H, Pyrazoline H_a), 3.82 (d, *J*=7.0 Hz, 1H, Pyrazoline H_b), 6.05 (dd, *J*=7.5 Hz, 1H, Pyrazoline H_c), 6.63–7.83 (m, *J*=7.5 Hz, 13H, Ar-H), 7.94 (s, 1H, CH-pyrrole), 9.94 (s, 1H, Ar-OH), 10.09 (s, 1H, NH-Ar), 10.69 (s, 1H, NH-Indole). FAB-MS (*m/z*): 397 $[M+1]^+$.

3-(4-Hydroxyphenyl)-5-(1H-indol-3-yl)-N-phenyl-4,5-dihydro-1H-pyrazole-1-carboxamide (4t): Pale yellow crystals, MF: $C_{24}H_{20}N_4O_2$; MW: 396; MP = 146–148 °C; % Yield: 68; R_f= 0.35 (4:6, Pet. ether: Ethyl acetate). Anal. Calcd for $C_{24}H_{20}N_4O_2$ (396): C, 72.71; H, 5.08; N, 14.13. Found: C, 72.78; H, 5.09; N, 1.16. IR (cm^{-1}): 3412 (NH), 3058–3112 (C-H aromatic), 2927–2839 (C-H aliphatic), 1685 (C=O), 1625 (C=N), 1416-1556 (C=C). ^{1}HNMR (DMSO-d_6): δ 3.34 (d, *J*=7.0 Hz, 1H, Pyrazoline Ha), 3.79 (d, *J*=7.0 Hz, 1H, Pyrazoline Hb), 6.32 (dd, *J*=7.5 Hz, 1H, Pyrazoline Hc), 6.73–7.86 (m, *J*=7.5 Hz, 13H, Ar-H), 7.98 (s, 1H, CH-pyrrole), 9.81 (s, 1H, Ar-OH), 10.07 (s, 1H, NH-Ar), 10.60 (s, 1H, NH-Indole). FAB-MS (m/z): 397 $[M+1]^+$.
